# Cognitive‐behavioural treatment for amphetamine‐type stimulants (ATS)‐use disorders

**DOI:** 10.1002/cl2.1026

**Published:** 2019-07-25

**Authors:** Takayuki Harada, Hiroshi Tsutomi, Rintaro Mori, David B Wilson

**Affiliations:** ^1^ Department of Psychology Mejiro University Tokyo Japan; ^2^ Faculty of International Relations University of Shizuoka Shizuoka Japan; ^3^ Department of Health Policy National Center for Child Health and Development Tokyo Japan; ^4^ Criminology, Law and Society George Mason University Fairfax Virginia USA

## Abstract

**Background:**

Amphetamine‐type stimulants (ATS) refer to a group of synthetic stimulants including amphetamine, methamphetamine, 3,4‐methylenedioxy‐methamphetamine (MDMA) and related substances. ATS are highly addictive and prolonged use may result in a series of mental and physical symptoms including anxiety, confusion, insomnia, mood disturbances, cognitive impairments, paranoia, hallucinations and delusion.

Currently there is no widely accepted treatment for ATS‐use disorder. However, cognitive‐behavioural treatment (CBT) is the first‐choice treatment. The effectiveness of CBT for other substance‐use disorders (e.g. alcohol‐, opioid‐ and cocaine‐use disorders) has been well documented and as such this basic treatment approach has been applied to the ATS‐use disorder.

**Objectives:**

To investigate the efficacy of cognitive‐behavioural treatment for people with ATS‐use disorder for reducing ATS use compared to other types of psychotherapy, pharmacotherapy, 12‐step facilitation, no intervention or treatment as usual.

**Search methods:**

We identified randomised controlled trials (RCT) and quasi‐RCTs comparing CBT for ATS‐use disorders with other types of psychotherapy, pharmacotherapy, 12 step facilitation or no intervention. We searched the Cochrane Drugs and Alcohol Group Specialised Register, Cochrane Central Register of Controlled Trials, MEDLINE via PubMed, Embase and five other databases up to July 2018. In addition, we examined reference lists of eligible studies and other systematic reviews. We contacted experts in the field.

**Selection criteria:**

Eligibility criteria consisted of RCTs and quasi‐RCTs comparing CBT versus other types of interventions with adult ATS users (aged 18 years or older) diagnosed by any explicit diagnostic system. Primary outcomes included abstinence rate and other indicators of drug‐using behaviours.

**Data collection and analysis:**

We used standard methodological procedures expected by Cochrane.

**Main results:**

Only two studies met the eligibility criteria. Both studies were at low risk of selection bias and reporting bias. In one study, almost half of participants in the intervention group dropped out and this study was at high risk of attrition bias. The studies compared a single session of brief CBT or a web‐based CBT to a waiting‐list control (total sample size across studies of 129). Results were mixed across the studies. For the single‐session brief CBT study, two out of five measures of drug use produced significant results, percentage of abstinent days in 90 days (odds ratio (OR) 0.22, 95% confidence interval (CI) 0.02 to 2.11) and dependence symptoms (standardised mean difference (SMD) –0.59, 95% CI–1.16 to–0.02). Little confidence could be placed in the results from this study give the small sample size (25 participants per group) and corresponding large CIs around the observed effects. For the web‐based CBT, there was no significant difference across different outcomes. Neither study reported adverse effects. The meta‐analytic mean across these two trials for drug use was not significant (SMD –0.28, 95% CI–0.69 to 0.14). In summary, overall quality of evidence was low and there was insufficient evidence to conclude that CBT is effective, or ineffective, at treating ATS use.

**Authors' conclusions:**

Currently, there is not enough evidence to establish the efficacy of CBT for ATS‐use disorders because of a paucity of high‐quality research in this area.

## 1 PLAIN LANGUAGE SUMMARY

### 1.1 Cognitive‐behavioural treatment for amphetamine‐type stimulants‐use disorders

#### 1.1.1 What was the aim of this review?

The aim of this Cochrane review was to find out whether cognitive‐behavioural treatment (CBT) is effective to treat people with amphetamine‐type stimulants (ATS)‐use disorders. Researchers in the Drugs and Alcohol Group of Cochrane collected and analysed all relevant studies to answer this question and found two studies.

#### 1.1.2 Key messages

The current evidence was inadequate to draw any firm evidence‐based treatment recommendations for the client population.

#### 1.1.3 What was studied in the review?

ATS are a group of synthetic stimulants and their use has been widespread globally. These types of drugs are highly addictive and prolonged use may result in a series of mental and physical symptoms including anxiety, confusion, insomnia (difficulty sleeping), mood disturbances, cognitive impairments (difficulty thinking and understanding), paranoia (irrational feeling that people are 'out to get you'), hallucinations (where someone experiences something that does not exist outside their own mind) and delusion (a mistaken belief).

Currently there is no widely accepted treatment for ATS‐use disorder. However, CBT is often the first choice of treatment. It is a psychological treatment (talking therapy) approach to modify distorted thoughts and beliefs, and maladaptive behaviours (things that people do to stop them from adjusting to situations). The effectiveness of CBT for other substance‐use disorders (e.g. alcohol‐, opioid‐ and cocaine‐use disorders) has been well documented and as such this basic treatment approach has been applied to the ATS‐use disorder. These types of therapies are expected to prevent relapse and decrease drug use.

#### 1.1.4 What are the main results of the review?

The review authors found two eligible studies. Both studies were conducted in Australia. One study compared a single session of brief CBT to a waiting‐list control where participants received no treatment during the study period. One study compared web‐based CBT to a waiting‐list control. Both studies were funded by the Australian Government of Health and Ageing.

The review showed that when participants received CBT, compared to waiting‐list control, there was no difference. There was insufficient evidence to conclude that CBT was effective or ineffective at treating ATS‐use disorders.

#### 1.1.5 How to up‐to‐date is this review

The review authors searched for studies that had been published up to July 2018.

## 2 BACKGROUND

### 2.1 Description of the condition

Amphetamine‐type stimulants (ATS) refer to a group of synthetic stimulants including amphetamine, methamphetamine and phenethylamines such as MDMA (3,4‐methylenedioxy‐methamphetamine) and its analogues. These substances have marked central and peripheral stimulant effects upon people and prolonged use results in a series of mental and physical symptoms that include anxiety, confusion, insomnia, mood disturbances, cognitive impairments, paranoia, hallucinations and delusion (Barr et al., 2006; Baylen & Rosenberg, 2006; Greene, Kerr, & Braitberg, 2008; Montoya, Sorrentino, Lukas, & Price, 2002; Morgan, 2000).

Since the 1990s, ATS use has been widespread globally and it is now the second most popular illicit drug in the world after cannabis. ATS use is of serious concern in East Asia, Southeast Asia, North America, Western Europe and Oceania (Farrell, Marsden, Ali, & Ling, 2002; UNODC, 2012). Statistics from the United Nations Office on Drugs and Crime (UNODC) indicate that approximately 25 million to 80 million people regularly use ATS worldwide (UNODC, 2012). Several new synthetic drugs have been gaining popularity, including MDMA and related amphetamines. These drugs are known as substituted amphetamines and they are characterised by enhanced hallucinogenic properties (Greene et al., 2008).

Amphetamines are highly addictive substances and produce euphoria and elevated mood. The short‐term adverse effects of amphetamines include high body temperature; cardiovascular system failure; hostility; irregular or increased heart rate; increased diastolic/systolic blood pressure; increased activity/talkativeness; euphoria; heightened sense of well‐being; decreased fatigue/drowsiness; decreased appetite; dry mouth; dilated pupils; increased respiration; heightened alertness/energy; nausea; headache; palpitations; altered sexual behaviour; tremor/twitching of small muscles; release of social inhibitions; and unrealistic feelings of cleverness, great competence and power (Barr et al., 2006; Lee & Rawson, 2008).

Amphetamines can be ingested, injected, smoked and snorted. Prolonged amphetamine use may result in more severe and devastating consequences. These include a series of mental and physical symptoms such as dizziness, mood or mental changes, chronic tiredness or weakness, physiological and behavioural disorders, flushed or pale skin, malnutrition, ulcers, repetitive motor activity, loss of co‐ordination and physical collapse, anxiety, confusion, insomnia, mood disturbances, cognitive impairments, paranoia, cardiac arrhythmias, toxic psychosis, amphetamine‐induced psychosis, convulsions, coma and death (Baylen & Rosenberg, 2006; Greene et al., 2008; Montoya et al., 2002).

ATS use is also related to infections of HIV/AIDS and other sexually transmitted diseases. The stimulating effects of ATS can impair judgement and inhibition, and lead people to engage in risky sexual behaviours. Moreover, sharing of injecting paraphernalia is common among people who inject drugs and such practice puts them at elevated risk of blood‐borne infectious diseases such as HIV, AIDS and hepatitis C (Degenhardt et al., 2010; Ellickson, McCaffrey, & Klein, 2009; King, Nguyen, Kosterman, Bailey, & Hawkins, 2012; Strathdee et al., 2010). Particularly, ATS use and associated HIV infections among men who have sex with men (MSM) poses a serious public health concern. More sexually adventurous MSM are likely to use ATS to increase sexual desire and make sexual intercourse less painful and more pleasurable. Thus, co‐occurring ATS use and unprotected risky sexual behaviours increase the risk of HIV and other sexually transmitted diseases (Thu Vu, Maher, & Zablotska, 2015).

The use of MDMA and its analogues is particularly prevalent among young people (UNODC, 2012). These drugs are usually taken orally as a tablet or capsule. Their pattern of use is different from that of 'traditional drugs.' Among young MDMA users, occasional use is most common, typically related to social events and involves the use of a relatively small amount of the drug. Users are likely to use multiple substances at the same time and MDMA tablets frequently contain other substances. Due to the combination of these substances, the consequences of MDMA use are unpredictable (Rogers et al., 2009).

MDMA and related substances have both stimulant and hallucinogenic effects. Therefore, the short‐term effects of these substances include increased heart rate and blood pressure, hyperactivity, euphoria, a heightened sense of well‐being, decreased fatigue/drowsiness and decreased appetite. In addition, distorted time and exaggerated sensory perception are frequently experienced. In contrast, long‐term consequences are not well known because abuse of these substances is relatively recent (Rogers et al., 2009). Young MDMA users frequently use drugs in club or all‐night dance parties, known as 'raves.' They tend to take drugs with alcohol and dance for a long time, and this may result in hyperthermia, dehydration, hypertension, and even kidney failure and death (NIDA, 2006).

ATS‐use disorder can be diagnosed by several set of criteria. For example, according to the ICD‐10 (International Classification of Diseases – 10th Revision), substance dependence syndrome is characterised by "a cluster of physiological, behavioural, and cognitive phenomena in which the use of a substance or a class of substances takes on a much higher priority for a given individual than other behaviours that once had greater value." Major diagnostic criteria include a strong desire to use, difficulties in controlling drug use, existence of withdrawal symptoms, evidence of tolerance, progressive neglect of alternative pleasures and persisting with substance use despite clear evidence of harmful consequences (WHO, 2004).

### 2.2 Description of the intervention

Currently there is no widely accepted treatment for ATS‐use disorder. This is especially the case for newly emerged ATS. However, cognitive‐behavioural treatment (CBT) is the first‐choice treatment (Lee & Rawson, 2008). The effectiveness of CBT for other substance‐use disorders (e.g. alcohol‐, opioid‐ and cocaine‐use disorders) has been well documented and as such this basic treatment approach has been applied to ATS‐use disorder. The treatment of MDMA use and the use of other new ATS drugs has not been extensively studied and the lack of evidence makes it difficult to know how best to treat people who use new ATS drugs (Rogers et al., 2009).

CBT for a substance‐use disorder can be defined as a structured approach to help clients reduce substance‐use behaviour by modifying their thoughts and behaviours. There are several therapies that are under the broad category of CBT, including behavioural therapy, cognitive therapy, CBY and the 'third‐wave' CBT. CBT usually employs a set of structured techniques such as motivational enhancement, relapse prevention, skills training, cognitive restructuring, stress management, emotional control and contingency management.

Moreover, CBT for substance‐use disorders has been used in many formats including individual therapy, group therapy and more recently computer‐based therapy.

### 2.3 How the intervention might work

CBT for a substance‐use disorder is based on the assumption that drug use is a learned behaviour and it emphasises individual commitment for recovery in order to learn new adaptive behaviours and ways of thinking. From the cognitive‐behavioural perspective, substance use is considered the result of coping deficits or maladaptive cognitions, or both. For example, if people do not have an appropriate coping repertoire or have positive outcome expectations towards substance use, or both, they are likely to use drugs in high‐risk situations (Marlatt & Witkiewits, 2005; Thombs, 2005). Therefore, coping skills training is considered an essential treatment component in CBT and emerging data suggested that acquisition and performance of skilful coping may account for CBT's effects on substance‐use disorders (Kiluk, Nich, Babuscio, & Carroll, 2010; Litt, Kadden, Cooney, & Kabela, 2003). CBT for substance‐use disorders is mainly designed to identify drug‐using triggers and provides people who use drugs with cognitive and behavioural skills to cope with these triggers to achieve and sustain abstinence from drugs. However, there is competing research which concludes that research currently failed to find solid evidence to explain CBT works through its effects on coping (Morgenstern & Longabaugh, 2000). Considering these findings, it is assumed that coping‐skills training may work through dynamic interaction between improvements of other important problem areas such as emotion and cognition (Marlatt & Witkiewits, 2005). Therefore, CBT also addresses thoughts, emotions, outcome expectations and lifestyles associated with drug use in order to address these multiple problem areas.

### 2.4 Why it is important to do this review

ATS use is increasing worldwide, especially in East Asia, Southeast Asia, North America, Western Europe and Oceania (Farrell et al., 2002; UNODC, 2012). Given this widespread ATS use, a comprehensive review of the effectiveness of treatment targeting ATS users is required to inform future research, clinical practice and policy making. Moreover, this review places a focus on CBT because CBT has multiple strengths over pharmacological treatment. For example, CBT is not associated with adverse effects and tends to have long‐lasting effects. Some studies indicated that ATS users who receive CBT reduce their ATS use even after treatment is terminated (Carroll et al., 2000; Rawson et al., 2002).

## 3 OBJECTIVES

To investigate the efficacy of cognitive‐behavioural treatment for people with ATS‐use disorder for reducing ATS use compared to other types of psychotherapy, pharmacotherapy, 12‐step facilitation, no intervention or treatment as usual.

## 4 METHODS

### 4.1 Criteria for considering studies for this review

#### 4.1.1 Types of studies

Randomised controlled trials (RCTs) and quasi‐RCTs.

#### 4.1.2 Types of participants

Adults (aged 18 years or older) with ATS dependence or abuse diagnosed by any set of criteria. This includes both the DSM‐IV (Diagnostic and Statistical Manual of Mental Disorders – Fourth Edition; APA, 2013) and ICD‐10 criteria as well as any other explicit ATS dependence or abuse diagnostic system. We also included studies where diagnosis relied solely on client self‐reporting of ATS dependence or abuse disorder without formal clinical assessment. We excluded people with comorbid conditions.

#### 4.1.3 Types of interventions

We included any CBT interventions in either individual or group therapy formats, in any treatment setting and any treatment modalities (e.g. face‐to‐face treatment, telephone treatment, computer‐based treatment). CBT interventions included behavioural therapy, cognitive therapy, CBT, 'third‐wave' CBT and any combinations of these therapies. However, we excluded any studies where CBT was delivered in conjunction with other types of psychotherapy and pharmacotherapy.

Comparison: other types of psychotherapy, pharmacotherapy, 12‐step facilitation (the intervention model to promote abstinence used in the self‐help groups), no intervention or treatment as usual.

#### 4.1.4 Types of outcome measures

**Primary outcomes**

- Abstinence rate measured by urine samples or self‐reported drug use, or both.
- Drug use measured as: amount of drug use, frequency of drug use, continuous using days or other measures of actual drug‐using behaviour. We only used measures of drug‐use behaviour within the past 30‐days or less.
- Dropout from treatment as measured as number of participants who did not complete the study protocol.

**Secondary outcomes**

- Overall mortality.
- Psychological variables such as self‐esteem and coping skills measured by standardised questionnaires (i.e. we only included psychological outcomes if they are based on a published measure that had been standardised or had known psychometric properties).
- Adverse outcomes.

### 4.2 Search methods for identification of studies

#### 4.2.1 Electronic searches

The Cochrane Drugs and Alcohol Group Information Specialist conducted systematic searches in the following databases for RCTs and controlled clinical trials without language, publication year or publication status restrictions.

- Cochrane Drugs and Alcohol Group Specialised Register (searched 2 July 2018) using the search strategy in Appendix 1;
- Cochrane Central Register of Controlled Trials (CENTRAL, the Cochrane Library, 2018, Issue 6) using the search strategy in Appendix 2;
- MEDLINE (PubMed) (1966 to 2 July 2018) using the search strategy in Appendix 3;
- Embase (embase.com) (1974 to 2 July 2018) using the search strategy in Appendix 4;
- Web of Science (1991 to 7 2 July 2018) using the search strategy in Appendix 5;
- PsycINFO (1985 to 2 July 2018) using the search strategy in Appendix 6.

The Information Specialist modelled subject strategies for databases on the search strategy designed for MEDLINE (PubMed). Where appropriate, they were combined with subject strategy adaptations of the highly sensitive search strategy designed by Cochrane for identifying RCTs and controlled clinical trials (as described in the *Cochrane Handbook for Systematic Reviews of Interventions* Chapter 6; Lefebvre, Manheimer, & Glanville, 2011).

We searched the following trials registries on 2 July 2018:

- ClinicalTrials.gov (www.clinicaltrials.gov);
- World Health Organization (WHO) International Clinical Trials Registry Platform (ICTRP) (apps.who.int/trialsearch/).

#### 4.2.2 Searching other resources

We contacted trial authors for additional trials and data. We also examined the reference lists of eligible studies and other systematic reviews for trials that may have otherwise been missed.

### 4.3 Data collection and analysis

#### 4.3.1 Selection of studies

Two review authors (TH & HT) independently screened the abstracts of all studies obtained through the search process and resolved any disagreements by discussion. Subsequently, we retrieved full‐text copies of all potentially relevant studies and two review authors (TH & HT) independently assessed the eligibility for inclusion. We resolved any disagreements by discussion and, when necessary, with a third review author (RM).

#### 4.3.2 Data extraction and management

At least two review authors (TH & HT) extracted the data using a predesigned data extraction form including a study design, characteristics of participants, treatment, control condition, funding source and outcomes. We resolved discrepancies through discussion or, if required, we consulted a third review author (RM). We entered data into Review Manager 2011 and check them for accuracy.

When information regarding any of the above was unclear, we attempted to contact trial authors to provide further details.

#### 4.3.3 Assessment of risk of bias in included studies

Two review authors (TH & HT) independently assessed risk of bias for each trial using the criteria outlined in the *Cochrane Handbook for Systematic Reviews of Interventions* (Higgins & Green, 2011). We resolved any disagreement by discussion or by involving a third review author RM). The recommended approach for assessing risk of bias in studies included in a Cochrane Review is a two‐part tool, addressing seven specific domains namely sequence generation and allocation concealment (selection bias), blinding of participants and providers (performance bias), blinding of outcome assessor (detection bias), incomplete outcome data (attrition bias), selective outcome reporting (reporting bias) and other sources of bias. The first part of the tool involves describing what was reported to have happened in the trial. The second part of the tool involves assigning a judgement relating to the risk of bias for that entry, in terms of low, high or unclear risk. To make these judgements, we used the criteria indicated by Higgins & Green, 2011, but adapted them to the addiction field. See Appendix 7 for details.

#### 4.3.4 Measures of treatment effect

##### 4.3.4.1 Dichotomous data

For dichotomous data, we presented results as summary odds ratio (OR) with a 95% confidence interval (CI).

##### 4.3.4.2 Continuous data

For continuous data, we used the standardised mean difference (SMD) effect size (Hedges' g). Ideally, these effect sizes are based on means, standard deviations (SD) and sample sizes for each condition. However, this effect size can be computed from a range of reported statistical information such as from a t‐test, P‐value from a t‐test, F‐test, regression coefficients, etc. Using this effect size index enables the combination of effect sizes across trials that examine a common construct but measure that construct differently.

#### 4.3.5 Unit of analysis issues

The unit of analysis was the individual participant.

##### 4.3.5.1 Cluster‐randomised controlled trials

We intended to include cluster‐RCTs in the analyses along with individual RCTs, if we identified any such studies. We intended to adjust their sample sizes using the methods described in Higgins & Green, 2011 using an estimate of the intracluster correlation coefficient (ICC) derived from the trial (if possible), from a similar trial or from a study of a similar population. If we use ICCs from other sources, we reported this and conducted sensitivity analyses to investigate the effect of variation in the ICC. If we identified both cluster‐RCTs and individual RCTs, we planned to synthesise the relevant information. We considered it reasonable to combine the results from both unless there was non‐negligible heterogeneity between the trial designs and the interaction between the effect of intervention and the choice of randomisation unit was considered likely.

#### 4.3.6 Dealing with missing data

For all outcomes, we carried out analyses, as far as possible, on an intention‐to‐treat basis (i.e. we attempted to include all participants randomised to each group in the analyses, and we analysed all participants in the group to which they were allocated, regardless of whether or not they received the allocated intervention). The denominator for each outcome in each trial was the number randomised minus any participants whose outcomes were missing.

#### 4.3.7 Assessment of heterogeneity

We assessed statistical heterogeneity in each meta‐analysis using the T^2^, I^2^ and Chi^2^ statistics. We regarded heterogeneity as substantial if the I^2^ statistic was greater than 50% or if the P value for the Chi^2^ test for heterogeneity was less than 0.10.

#### 4.3.8 Assessment of reporting biases

If there were 10 or more included trials in the meta‐analysis, we intended to investigate reporting biases (such as publication bias) using funnel plots and the trim‐and‐fill method. Also, we planned to assess asymmetry visually using funnel plots. However, the review included only two trials and the assessment was not performed.

#### 4.3.9 Data synthesis

We carried out statistical analysis using Review Manager 2011. We used a random‐effects model for combining data as the assumptions of the fixed‐effect model were unreasonable for this literature. Trials are likely to differ in numerous ways that may affect the underlying treatment effect being estimated such as the specifics of the CBT being implemented, the context of the treatment and the unique characteristics of the population. The random‐effects model converges on the fixed‐effect model as the data become homogeneous, so this approach is reasonable and consistent with recommended practice within the meta‐analysis literature. We presented the results as the mean treatment effect with its 95% CI.

##### 4.3.9.1 'Summary of findings' table

We assessed the overall quality of the evidence for the primary outcomes (abstinence rate, drug use, and treatment drop‐out) and adverse effects using the GRADE system and presented them in a 'Summary of findings' table (Atkins et al., 2004). The GRADE system uses the following criteria for assigning grade of evidence.

- High: we are very confident that the true effect lies close to that of the estimate of the effect.
- Moderate: we are moderately confident in the effect estimate: the true effect is likely to be close to the estimate of the effect, but there is a possibility that it is substantially different.
- Low: our confidence in the effect estimate is limited: the true effect may be substantially different from the estimate of the effect.
- Very low: we have very little confidence in the effect estimate: the true effect is likely to be substantially different from the estimate of effect.

The grade of evidence was decreased if there was:

- serious (–1) or very serious (–2) limitation to study quality;
- serious (–1) or very serious (–2) inconsistency between study results;
- some (–1) or major (–2) uncertainty about directness (the correspondence between the population, the intervention, or the outcomes measured in the studies actually found and those under consideration in our systematic review);
- serious (–1) or very serious (–2) imprecision of the pooled estimate;
- publication bias strongly suspected (–1).

#### 4.3.10 Subgroup analysis and investigation of heterogeneity

If we identified substantial heterogeneity, we planned to investigate it using subgroup analyses and sensitivity analyses.

We intended to carry out the following subgroup analyses:

- gender;
- age such as minor or adult;
- treatment duration such as brief intervention or more lengthy intervention;
- treatment setting such asresidential or community‐based;
- characteristics of the CBT treatment such as presence or absence of the following treatment component: relapse prevention, motivational component, contingency management, cognitive restructuring and social skills training;
- characteristics of treatment providers such as inhouse therapists or outside contractors;
- characteristics of CBT therapists such as length of training.

We planned to use the following outcomes in subgroup analysis:

- abstinence rate measured by urine samples or self‐report, or both.

We also planned to perform these moderator analyses using random‐effects meta‐analytic regression methods or analogue‐to‐the ANOVA, depending on the nature of the moderator variable. We planned to use Stata for these analyses using macros developed by one review author (DW) and available at mason.gmu.edu/~dwilsonb/ma.html.

However, neither subgroup analysis nor sensitivity analysis was carried out because the review included only two RCTs.

#### 4.3.11 Sensitivity analysis

We planned to carry out sensitivity analysis to explore the effects of trial quality assessed by allocation concealment and other risk of bias components such as attrition bias by omitting trials at high risk of bias for these components. We planned to restrict sensitivity analysis to the primary outcome.

## 5 Results

### 5.1 Description of studies

See: Characteristics of included studies; Characteristics of excluded studies tables.

#### 5.1.1 Results of the search

**Figure 1:**
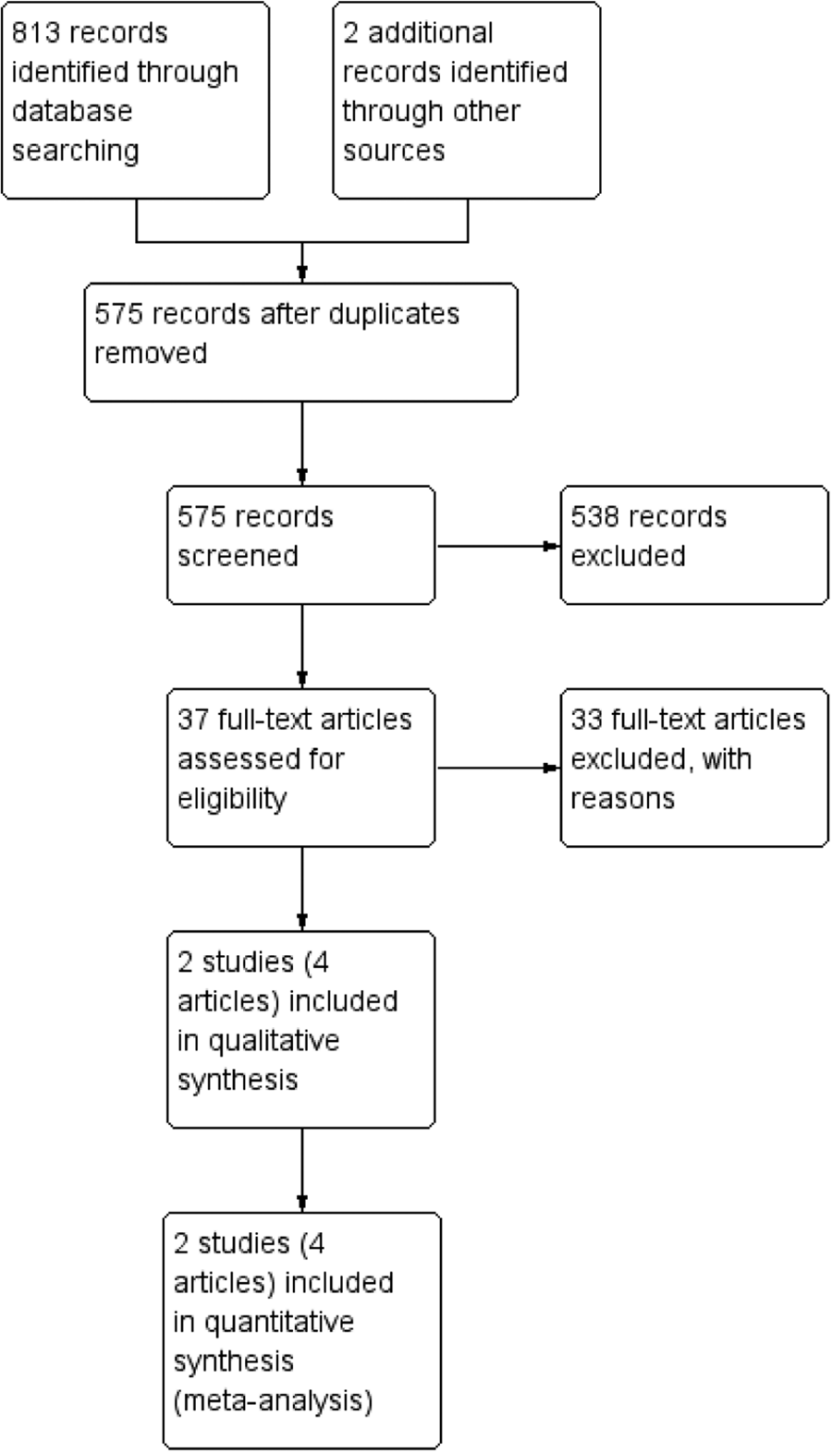
Study flow diagram

We retrieved 813 records through database searching. Further handsearching and personal communication identified an additional two studies. Once duplicates were removed, there were 575 records. We excluded 538 records based on titles and abstracts. We assessed 37 full texts for eligibility and excluded 33 references, with reasons (see Characteristics of excluded studies table). As the result of the eligibility check, only two studies (three articles) were finally included (Martin 2010; Tait et al., 2015). For a further description of our screening process, see the PRISMA study flow diagram (Figure 1).

#### 5.1.2 Included studies

We identified only two trials (Martin 2010; Tait et al., 2015). Martin 2010 conducted an RCT with 50 MDMA users, who were randomly assigned either to a single‐session brief CBT or a waiting‐list control (assessment‐only three‐month delayed treatment condition). Participants were 50 adult MDMA users (mean age: 28.5 (SD 9.2) years), 31 men (62%) and 19 women (38%). Study was conducted in Australia and 68% of participants were born in Australia. Polydrug use was common such as alcohol, cannabis, other types of amphetamines and cocaine. The main components of the intervention included an assessment, personalised feedback and optional skills training. Tait et al. (2015) compared a web‐based CBT to a waiting‐list control. Participants were 160 ATS users, 81 (mean age: 22.2 (SD 5.5)) were assigned to the intervention group and 79 (mean age: 22.5 (SD 7.1)) to the control group. All were Australian residents. There were 64 (79%) men in the intervention group and 57 (72%) men in the control group. All study procedures were undertaken via the Internet including enrolment, screening and treatment.

#### 5.1.3 Excluded studies

The reasons for exclusion varied. Eleven studies compared CBT versus other type of CBT and 11 studies included participants with other substance use problems (including cocaine, opiates, alcohol, etc.) but not reported separate outcomes data for ATS users, five articles were secondary publication of already excluded studies, one study was not an RCT and in two studies the comparison intervention were not clearly described. We asked study authors for ATS users only data, all but one failed to provide the data. See Characteristics of excluded studies table for details.

### 5.2 Risk of bias in included studies

**Figure 2:**
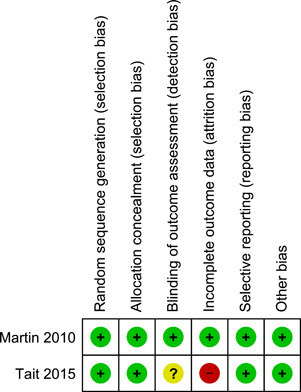
Risk of bias summary: review authors' judgements about each risk of bias item for each included study

**Figure 3:**
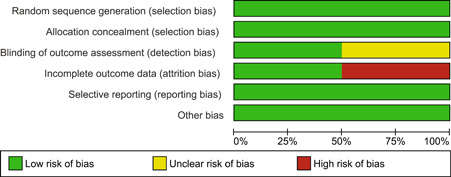
Risk of bias graph: review authors' judgements about each risk of bias item presented as percentages across all included studies

The details of the risk of bias assessment are given in the 'Risk of bias' table (Figure 2; Figure 3).

#### 5.2.1 Allocation (selection bias)

Martin 2010 used an independent researcher who prepared a randomisation sequence contained in sealed envelopes. Tait et al. (2015) conducted the randomisation process using a fully automated system (Tait et al., 2015). It seemed that both studies performed allocation appropriately and concealed it from both researchers and participants. Therefore, selection bias was unlikely in either study.

#### 5.2.2 Blinding (performance bias and detection bias)

**Performance bias:** blinding of personnel delivering the intervention and participants is not feasible. It is not desirable to blind participants to the knowledge of which condition they are in. Knowledge that you are participating in a cognitive‐behavioural programme is part of the intervention (this knowledge is often categorised along with other non‐specific intervention factors). For this reason, we did not assess the risk of bias of this item.

**Detection bias:** Martin 2010 used an independent researcher to collect outcome measures and it seemed that blinding was appropriately done. Tait et al. 2015 was at unclear risk because there was no information to permit judgement.

#### 5.2.3 Incomplete outcome data (attrition bias)

Martin 2010 had balanced missing outcome data across the groups and used an intention‐to‐treat analysis; therefore, we judged the study at low risk of bias. In Tait et al. 2015, almost half of the participants allocated to the intervention group did not receive all the modules of the intervention and 53% of the participants were lost to follow‐up. In contrast, all control participants received the intended condition of no intervention but 48% were lost to follow‐up. The proportion of missing outcomes was large although ITT analysis was performed based on participants with baseline data plus at least one follow‐up. We judged the study at high risk of bias.

#### 5.2.4 Selective reporting (reporting bias)

Tait et al. 2015 published the study protocol prior to the report (see Tait et al., 2012 under Tait et al., 2015) and reported all prespecified outcomes in the final paper. Martin 2010 provided no predetermined protocol for the trial and we had no information on selective reporting. However, of the five measures of ATS use report, three were statistically not significant suggesting that the authors did not censor outcomes based on statistical significance. For these reasons, we judged both studies at low risk of reporting bias.

#### 5.2.5 Other source of bias

Both studies appear to be free of other sources of bias.

### 5.3 Effects of interventions

#### 5.3.1 Single‐session motivational and cognitive‐behavioural treatment

We found one trial comparing single‐session multi‐component CBT versus waiting‐list control (Martin 2010).

##### 5.3.1.1 Abstinence rate

The percentage of 90‐day MDMA abstinence was over four times greater in the treated participants, although this difference was not significant (OR 0.22, 95% CI 0.02 to 2.11; 50 participants). See Analysis 1.1.

##### 5.3.1.2 Drug use

There were significant effects favouring the treated participants for the number of dependence symptoms reported (SMD –0.59, 95% CI–1.16 to–0.02; 50 participants) and the score of the Severity of Dependence Scale (SDS) (SMD –0.62, 95% CI–1.18 to–0.05; 50 participants). The days of ecstasy use on the past 90 days and the mean number of tablets used did not differ between CBT and waiting‐list control (days of ecstasy use: SMD –0.45, 95% CI–1.04 to 0.09; mean tablets used: –0.48, 95% CI–1.04 to 0.09; 50 participants). See Analysis 1.2; Analysis 1.3; Analysis 1.4; Analysis 1.5.

##### 5.3.1.3 Dropout from treatment

The study did not report dropouts because the control was waiting‐list control.

##### 5.3.1.4 Psychological variables

The study did not report psychological variables.

##### 5.3.1.5 Web‐based cognitive‐behavioural treatment

We found one trial comparing web‐based CBT versus waiting‐list control (Tait et al., 2015).

##### 5.3.1.6 Abstinence rate

The study did not report abstinence rate.

##### 5.3.1.7 Drug use

Tait et al. 2015 measured ATS use using the self‐report Alcohol, Smoking, Substance Involvement Screening Test (ASSIST). The difference was not significant (SMD –0.05, 95% CI–0.49 to 0.39; 160 participants; Analysis 2.2). However, this measure excluded ATS drug use.

##### 5.3.1.8 Dropout from treatment

The study did not report dropouts because the control was waiting‐list control.

##### 5.3.1.9 Psychological variables

There were no significant differences in any of the secondary measures. These outcomes included intended help seeking, actual help seeking, K‐10 score, days out of role, days part out of role and quality of life. See Analysis 2.3; Analysis 2.4; Analysis 2.5; Analysis 2.6; Analysis 2.7.

##### 5.3.1.10 Any cognitive‐behavioural treatment

**Figure 4:**
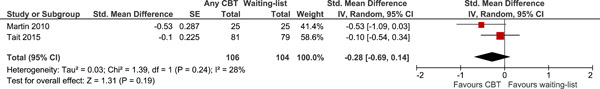
(Analysis 3.1) Forest plot of comparison: 3 Cognitive‐behavioural treatment (CBT) versus waiting‐list, outcome: 3.2 Drug use

We conducted a single meta‐analysis of any drug use across these two studies by using the primary outcome of ATS use from the Tait et al. 2015 and averaging the four continuous measures of drug use reported in Martin 2010. We did this given that Martin 2010 did not specify any of these four as a primary outcome. Furthermore, the effects are quite similar across these outcomes, ranging from –0.62 to–0.45. The mean effect for Martin 2010 was an SMD –0.53 and standard error of 0.288 (this was the mean standard error but these standard errors were essentially the same across the four effect sizes). The difference was not significant (SMD –0.28, 95% CI–0.69 to 0.14; 210 participants; Summary of findings table 1). See Analysis 3.1; Figure 4.

## 6 Discussion

### 6.1 Summary of main results

Despite extensive searches, only two trials met the eligibility criteria. This result reflects a paucity of RCTs in this area. The purpose of this review was to compare CBT to other types of treatment; however, such RCTs were especially rare. For the treatment for ATS‐use disorders, CBT is frequently used and the use of other treatment approaches are rare. This may be because evidence of CBT for other 'traditional' substance‐use disorders has been documented.

Interventions conducted in included trials were uncommon. Usually, treatment for substance‐use disorders is delivered over numerous weeks and in a face‐to‐face format, sometimes in a group setting; on the contrary, Martin 2010 evaluated a single‐session brief intervention and Tait et al. 2015 evaluated a web‐based intervention.

Martin 2010 provided a multi‐component intervention in a single session including an assessment, personalised feedback within a motivational interviewing framework and relapse prevention skills training. Typical substance abuse treatment has more therapeutic components such as identification of triggers, coping skills training, emotional management, alternative activities, cognitive restructuring, support network building and lapse management. Because of the nature of the intervention, Martin 2010 left most of these components out.

Only two of the five effects (dependence symptoms, SDS score) were significant given the rather small sample size (25 in each group).

Tait et al. 2015 delivered an Internet‐based intervention comprised of three modules: exploration of problems associated to ATS use; the pros and cons of ATS use; and goal setting and behavioural change. All outcome measures used in the study were self‐reported and no urinalysis was conducted which is usually recommended in clinical trials of substance‐use disorders. This is one of the study limitations and there was no significant effect for the eight outcomes including ATS use, poly‐drug use and quality of life.

We identified only two CBT trials and both were 'uncommon' interventions. In usual clinical practice, face‐to‐face treatment with more, longer therapeutic contacts are common (Galloway et al., 2000; Knapp, Soares, Farrell, & Silva de Lima, 2008). However, there is a paucity of research to evaluate such common CBT interventions and existing RCTs are of relatively low quality. Therefore, we did not have enough evidence to determine whether CBT is effective.

In some excluded studies, people with cocaine use, opiate use or excessive alcohol use were study participants in addition to ATS users and their data could not be excluded. Other studies compared CBT to other types of CBT. These studies were excluded since the purpose of this review is to compare CBT to other types of treatment approaches. However, several trials found positive effects over CBT.

Also, some excluded studies compared CBT to other types of CBT, for example traditional CBT versus mindfulness‐based CBT and CBT versus CBT plus "ad‐on" interventions such as pharmacotherapy and contingency management. Other studies evaluated CBT targeting subgroup ATS users with specialised treatment needs such as women (Ruglass, Hien, Hu, & Campbell, 2014), MSM (Reback & Shoptaw, 2014; Santos et al., 2014; Shoptaw et al., 2008), people with co‐occurring disorders (Baker et al., 2006; Barrowclough et al., 2009; Beutler et al., 2003), and ethnic minorities (Witkiewitz, Greenfield, & Bowen, 2013). These studies found some positive results but were excluded from this review because they did not meet the eligibility criteria. Moreover, most studies were carried out in Western countries. Since ATS use has been spread globally, treatment approaches must be evaluated in a specific cultural and social background. A small number of studies were conducted in other areas such as Japan (Harada, 2010), Taiwan (Yen, Wu, Yen, & Ko, 2004), and Thailand (Suvanchot, Somrongthong, & Phukhao, 2012), and there were some positive results. However, these studies were not included because they did not fulfil the eligibility criteria.

### 6.2 Overall completeness and applicability of evidence

The review included only two trials and the results found no clear evidence of the benefits of CBT for ATS‐use disorders. Both of the included studies examined relatively uncommon approaches such as a one‐session brief therapy and a web‐based therapy. Therefore, the evidence obtained in this review could not be applicable across the wide range of treatment modalities and settings.

### 6.3 Quality of the evidence

There were only two studies included in the meta‐analysis. Moreover, there were several methodological flaws in the included studies such as possible incomplete blinding, a very small sample size and a large loss to follow‐up in one study. Also, Martin 2010 provided no treatment fidelity data and this could pose a concern. These limitations result in performance bias, attrition bias and imprecision of results. Therefore, overall quality of evidence was low.

### 6.4 Potential biases in the review process

We identified no potential biases.

### 6.5 Agreements and disagreements with other studies or reviews

We found two systematic reviews of psychological treatment for methamphetamine dependence (Ciketic, Hayatbakhsh, Doran, Najman, & McKetin, 2012; Lee 2008). One was a systematic review of CBT for methamphetamine dependence, which included 12 studies and concluded that CBT and contingency management are effective for the condition (Lee 2008). The review raised a different question from ours and included studies comparing different types of CBT (e.g. CBT versus CBT plus contingency management). The other systematic review included both psychological and pharmacological interventions (Ciketic et al., 2012). They suggested that psychosocial interventions including CBT, contingency management, relapse prevention and other behavioural therapies are promising treatment options. Neither review conducted a meta‐analysis or calculated an effect size. These reviews supported the effectiveness of CBT but in our review included only two studies and there was insufficient evidence because of a paucity of research comparing CBT and other types of psychosocial interventions.

We found another Cochrane Review, "Psychosocial interventions for psychostimulant misuse" (Minozzi, Saulle, De Crescenzo, & Amato, 2016). The scope of the review was broader than ours in terms of the intervention and the condition. The review was not limited to CBT and included interventions such as interpersonal therapy, 12‐step facilitation, psychodynamic therapy and drug counselling. Most participants were cocaine users and only six of 52 included studies were with ATS users. The review concluded that psychosocial interventions, when compared to no intervention, reduced the dropout rate, increased continuous abstinence at the end of treatment and increased the longest period of abstinence. However, compared to treatment as usual, the dropout rate was significantly reduced but no significant changes in other outcomes.

We found several literature reviews. Baker 2003 reported that CBT and contingency management were effective approaches; however, evidence was very limited because of a paucity of well‐conducted controlled studies. Another review, Vocci & Montoya, 2009, concluded that psychological interventions such as CBT and contingency management were moderately effective in achieving abstinence for amphetamine and cocaine users.

In summary, we found several similar reviews and they all agreed that CBT and contingency management seem to be effective. However, evidence was weak because existing RCTs were limited and the research quality was relatively low.

## 7 Authors' conclusions

### 7.1 Implications for practice

Currently, there is insufficient evidence to support the efficacy of cognitive‐behavioural treatment (CBT) for amphetamine‐type stimulant (ATS)‐use disorders.

### 7.2 Implications for research

More randomised trials are required to establish evidence for CBT for ATS‐use disorders, especially CBT should be compared to other types of treatment options, no treatment and treatment as usual without a CBT component. Moreover, more trials are necessary targeting newly emerged ATS users and subgroups of participants who have specific treatment needs including women, adolescents, older people, men who have sex with men, non‐Westerners and people with comorbid conditions.

## Contributions of authors

TH: protocol development, study search and selection, contact with study authors, risk of bias assessment, interpretation of data, providing clinical perspective.

HT: protocol development, study selection, risk of bias assessment, interpretation of data, providing methodological perspective.

RM: protocol development, interpretation of data, providing general advice on the review.

DW: protocol development, statistical analysis, interpretation of data, results and discussion, providing methodological and clinical perspective.

## Declarations of interest

TH: none known.

HT: none known.

RM: none known.

DW: none known.

## Differences between protocol and review

The original inclusion criteria were set as trials involving participants with ATS‐use disorder diagnosed by any set of formal criteria such as DSM and ICD were included. However, studies that rely solely on client self‐report of an ATS dependence or use were also included in the review. In real‐world clinical settings, many clients receive CBT without any formal diagnosis. Even formal diagnostic criteria heavily rely on patients' self‐report including the amount of use, days of use, and existence of craving and subjective withdrawal symptoms (APA, 2013). Thus, self‐reported drug use might be the optimal form of data because any subjective analysis such as hair and urine analysis cannot provide full data on past drug use (Parrott, 2000). Given such clinical significance, we changed the inclusion criteria on study participants. Moreover, we included treatment dropout rate in primary outcomes because it is clinically significant outcome in addiction treatment.

**Published notes**

**Characteristics of studies**

**Characteristics of included studies**

**Martin 2010**

**Table:** 

**Methods**	Pilot RCT
**Participants**	50 non‐treatment seeking adults who used MDMA at least once in the past month without severe cognitive impairment.
	Mean age: 28.5 (SD 9.2) years
	Gender: 31 men (62%) and 19 women (30%)
	Poly‐drug use was common among participants (alcohol, cannabis, amphetamine and cocaine).
**Interventions**	**Intervention:** single‐session multi‐component CBT including an assessment, personalised feedback and relapse prevention skills training (25 participants). The intervention was annualised and delivered by a doctoral level clinician. However, no fidelity data were available.
	**Control:** waiting‐list control (25 participants).
**Outcomes**	Primary outcomes: frequency of use, quantity of use and number of DSM‐IV symptoms
**Notes**	Country: Australia
	Funding: Australian Government of Health and Ageing
	Declaration of interest: no conflicts of interest

**Risk of bias table**

**Table:** 

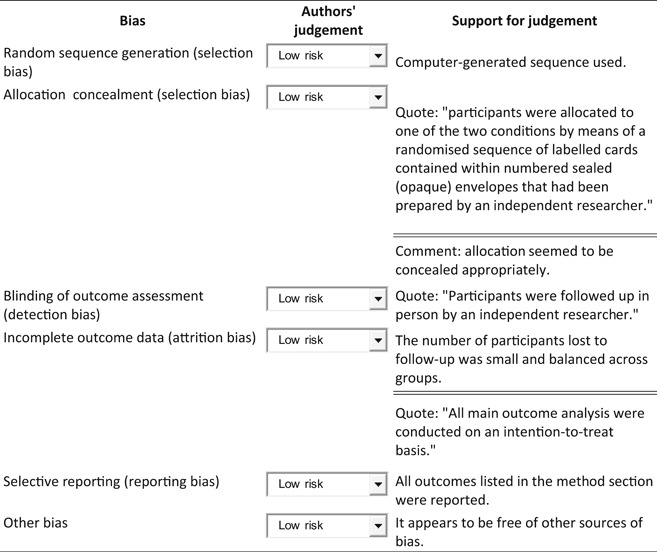

**Tait 2015**

**Table:** 

**Methods**	RCT comparing a web‐based CBT to a waiting‐list control
**Participants**	160 adults who reported ATS use in last 3 months
	Mean age: intervention: 22.2 (SD 5.5) years; control: 22.5 (SD 7.1) years
	Gender: intervention: 64 (79%) men; control: 57 (72%) men
**Interventions**	**Intervention:** web‐based CBT intervention with 3 modules (81 participants)
	**Control:** waiting‐list control (79 participants)
**Outcomes**	Self‐reported ATS use, quality of life, psychological distress, day out of role, poly‐drug use, general help‐seeking intentions, actual help‐seeking and readiness to change
**Notes**	Country: Australia
	Funding: Australian Government of Health and Ageing, NHMRC Fellowship, Curtin University Research Fellowship
	Declaration of interest: no conflicts of interest

**Risk of bias table**

**Table:** 

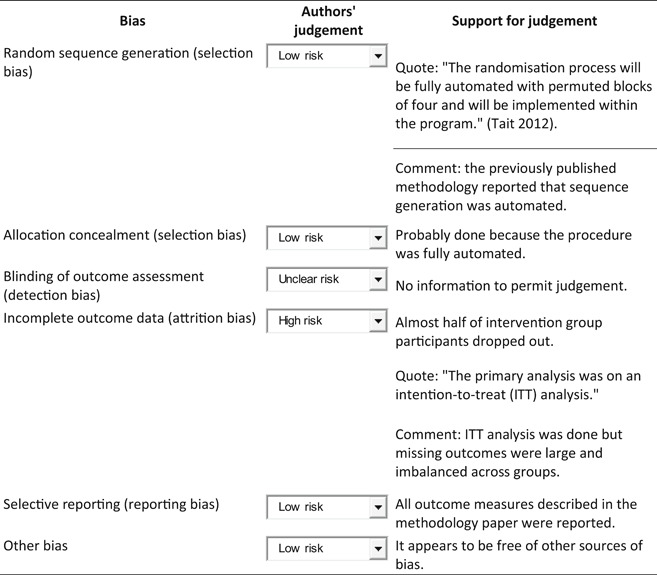

ATS: amphetamine‐type stimulant; CBT: cognitive‐behavioural treatment; DSM‐IV: Diagnostic and Statistical Manual of Mental Disorders – Fourth Edition; ITT: intention to treat; MDMA: 3,4‐methylenedioxy‐methamphetamine; NHMRC: National Health and Medical Research Council; RCT: randomised controlled trial; SD: standard deviation.

### Characteristics of excluded studies

**Baker 2001**

**Table:** 

**Reason for exclusion**	Participants in both groups used same therapy material.

**Baker 2005**

**Table:** 

**Reason for exclusion**	Enlarged study of Baker 2001.

**Baker 2006**

**Table:** 

**Reason for exclusion**	Participants with other drug use included and their data could not be excluded.

**Barrowclough 2009**

**Table:** 

**Reason for exclusion**	Participants with other drug use included and their data could not be excluded.

**Beutler 2003**

**Table:** 

**Reason for exclusion**	Other drug users included and their data could be excluded from treatment group but not from control group.

**Brooks 2003**

**Table:** 

**Reason for exclusion**	Participants with other drug use included and their data could not be excluded.

**Glasner‐Edwards 2017**

**Table:** 

**Reason for exclusion**	Participants with other drug use included and their data could not be excluded.

**Herrell 2000**

**Table:** 

**Reason for exclusion**	Another report of Rawson 2004.

**Jaffe 2007**

**Table:** 

**Reason for exclusion**	CBT compared to another type of CBT.

**Kay‐Lambkin 2010**

**Table:** 

**Reason for exclusion**	CBT compared to another type of CBT.

**Kay‐Lambkin 2011**

**Table:** 

**Reason for exclusion**	Another report of Baker 2005.

**Keoleian 2013**

**Table:** 

**Reason for exclusion**	Preliminary randomised cross‐over study that compare same intervention with different order.

**Ling 2014**

**Table:** 

**Reason for exclusion**	Combined therapy (CBT + pharmacotherapy) compared to placebo.

**Marinelli‐Casey 2008**

**Table:** 

**Reason for exclusion**	Another report of Rawson 2004.

**Mausbach 2007**

**Table:** 

**Reason for exclusion**	Focus of intervention was to reduce high‐risk sexual behaviours and drug use outcome not measured.

**McDonell 2013**

**Table:** 

**Reason for exclusion**	Participants with other drug use included and their data could not be excluded.

**Nyamathi 2017**

**Table:** 

**Reason for exclusion**	CBT compared to another type of CBT.

**Peck 2005**

**Table:** 

**Reason for exclusion**	CBT compared to another type of CBT.

**Peirce 2006**

**Table:** 

**Reason for exclusion**	Participants with other drug use included and their data could not be excluded.

**Petry 2005**

**Table:** 

**Reason for exclusion**	Cocaine users included and their data could not be excluded.

**Rawson 2004**

**Table:** 

**Reason for exclusion**	CBT compared to TAU controls but several TAU conditions contain CBT components.

**Rawson 2006**

**Table:** 

**Reason for exclusion**	Most participants were cocaine users and their data could not be excluded. CBT compared to another type of CBT.

**Roll 2006**

**Table:** 

**Reason for exclusion**	CBT compared to another type of CBT.

**Rosenblum 2005**

**Table:** 

**Reason for exclusion**	CBT compared to another type of CBT.

**Ruglass 2014**

**Table:** 

**Reason for exclusion**	Participants were not diagnosed by explicit diagnostic criteria. Participants with other drug use included and their data could not be excluded.

**Santos 2014**

**Table:** 

**Reason for exclusion**	Participants with other drug use included and their data could not be excluded.

**Shoptaw 2005**

**Table:** 

**Reason for exclusion**	CBT compared to another type of CBT (CBT vs contingency management).

**Shoptaw 2008**

**Table:** 

**Reason for exclusion**	CBT compared to another type of CBT (CBT vs Social Skills Training).

**Sitharthan 1999**

**Table:** 

**Reason for exclusion**	Participants with other drug use included.

**Smout 2010**

**Table:** 

**Reason for exclusion**	CBT compared to another type of CBT.

**Suvanchot 2012**

**Table:** 

**Reason for exclusion**	Not an RCT.

**Witkiewitz 2013**

**Table:** 

**Reason for exclusion**	CBT compared to another type of CBT (mindfulness‐based therapy).

**Yen 2004**

**Table:** 

**Reason for exclusion**	Details of comparison treatment unclear.

CBT: cognitive‐behavioural treatment; RCT: randomised controlled trial; TAU: treatment‐as‐usual.

**Characteristics of studies awaiting classification**

**Characteristics of ongoing studies**

**Summary of findings tables**

**1 Any cognitive‐behavioural treatment compared to waiting‐list control for amphetamine‐type stimulant‐use disorders**

**Table:** 

**Any CBT compared to waiting‐list control for ATS‐use disorders**						
**Patient or population:** adults with ATS‐use disorders						
**Setting:** community						
**Intervention:** CBT						
**Comparison:** waiting‐list control						
**Outcomes**	Anticipated absolute effects* (95% CI)		**Relative effect (95% CI)**	**№ of participants (studies)**	**Quality of the evidence (GRADE)**	**Comments**
	**Risk with waiting‐list control**	**Risk with any CBT**				
**Drug use**	—	SMD 0.28 lower	—	210	**Low** ^a^	—
		(0.69 lower to 0.14 higher)		(2 studies)	⊕⊕⊝⊝	
*The risk in the intervention group (and its 95% confidence interval) is based on the assumed risk in the comparison group and the **relative effect** of the intervention (and its 95% CI).						
**ATS:** amphetamine‐type stimulant; **CBT:** cognitive‐behavioural treatment; **CI:** confidence interval; **SMD:** standardised mean difference.						
**GRADE Working Group grades of evidence**						
**High quality:** we are very confident that the true effect lies close to that of the estimate of the effect						
**Moderate quality:** we are moderately confident in the effect estimate: the true effect is likely to be close to the estimate of the effect, but there is a possibility that it is substantially different						
**Low quality:** our confidence in the effect estimate is limited: the true effect may be substantially different from the estimate of the effect						
**Very low quality:** we have very little confidence in the effect estimate: the true effect is likely to be substantially different from the estimate of effect						

^a^Quality downgraded two levels because of limitations in the design and implementation of included studies (blinding and attrition) and imprecision of results (small sample size).

## Other published versions of this review

### Classification pending references

#### Data and analyses

**1 Single‐session motivational and cognitive‐behavioural treatment (CBT) versus waiting‐list control**

**Table:** 

Outcome or Subgroup	Studies	Participants	Statistical Method	Effect Estimate
1.1 Abstinent rate (% at 90 days)	1	50	Odds Ratio (IV, Random, 95% CI)	0.22 [0.02, 2.11]
1.2 Drug use: days ecstasy use past 90	1	50	Std. Mean Difference (IV, Random, 95% CI)	−0.45 [−1.01, 0.12]
1.3 Drug use: mean tablets used	1	50	Std. Mean Difference (IV, Random, 95% CI)	−0.48 [−1.04, 0.09]
1.4 Psychological variables: dependence symptoms	1	50	Std. Mean Difference (IV, Random, 95% CI)	−0.59 [−1.16, −0.02]
1.5 Psychological variables: severity of Dependence Scale score	1	50	Std. Mean Difference (IV, Random, 95% CI)	−0.62 [−1.18, −0.05]

**2 Web‐based cognitive‐behavioural treatment (CBT) versus waiting‐list control**

**Table:** 

Outcome or Subgroup	Studies	Participants	Statistical Method	Effect Estimate
2.1 Drug use: amphetamine‐type stimulant use	1	160	Std. Mean Difference (IV, Random, 95% CI)	−0.10 [−0.54, 0.34]
2.2 Drug use	1	160	Std. Mean Difference (IV, Random, 95% CI)	−0.05 [−0.49, 0.39]
2.3 Psychological variables: intended help‐seek	1	160	Std. Mean Difference (IV, Fixed, 95% CI)	0.31 [−0.13, 0.75]
2.4 Psychological variables: K‐10 score	1	160	Std. Mean Difference (IV, Fixed, 95% CI)	−0.10 [−0.54, 0.34]
2.5 Psychological variables: days out of role	1	160	Std. Mean Difference (IV, Random, 95% CI)	0.02 [−0.42, 0.46]
2.6 Psychological variables: days part out of role	1	160	Std. Mean Difference (IV, Random, 95% CI)	−0.05 [−0.49, 0.39]
2.7 Psychological variables: quality of life	1	160	Std. Mean Difference (IV, Random, 95% CI)	−0.19 [−0.63, 0.25]

**3 Any cognitive‐behavioural treatment (CBT) versus waiting‐list control**

**Table:** 

Outcome or Subgroup	Studies	Participants	Statistical Method	Effect Estimate
3.1 Drug use	2	210	Std. Mean Difference (IV, Random, 95% CI)	−0.28 [−0.69, 0.14]

## Sources of support

### Internal sources

 

- No sources of support provided

 

### External sources

- Health Labour and Sciences Research Grant, Ministry of Health, Labour and Sciences, Japan
- Japan Agency for Medical Research and Development, Japan

## Supporting information

Supporting information
