# Supplementary material for: Cognitive‐behavioural treatment for amphetamine‐type stimulants (ATS)‐use disorders
Source: Campbell Syst Rev. 2019 Jul 25;15(1-2):e1026. doi: 10.1002/cl2.1026 (PMC8356519; doi:10.1002/cl2.1026)
Supplement: Supplementary file 1 — Supporting information [file CL2-15-e1026-s001.docx]

**Appendices**

**1 Cochrane Drug and Alcohol Group Specialised Register search strategy**

**CDAG Specialised register (via CRSLive)**

**2 July 2018 (52 hits)**

#1 (((amphetamine OR amfetamine OR methamphetamine OR mdma OR ecstasy OR dextroamphetamine OR stimulant OR stimulants):xdi)) AND ( INREGISTER)

#2 (((amphetamine OR amfetamine OR methamphetamine OR mdma OR ecstasy OR dextroamphetamine OR stimulant OR stimulants):ti)) AND ( INREGISTER)

#3 ((cbt:ti OR cbt:ab OR cbt:xin)) AND ( INREGISTER)

#4 (((cogniti* NEAR3 (behavio* OR therap*)))) AND ( INREGISTER)

#5 (((cogniti* NEAR3 (technique* OR restructur* OR challeng*)))) AND ( INREGISTER)

#6 #3 OR #4 OR #5

#7 #1 OR #2

#8 #6 AND #7

**2 CENTRAL search strategy**

**CENTRAL (via onlinelibrary.wiley.com)**

**Issue 6, 2018 (118 hits)**

#1 MeSH descriptor: [Cognitive Therapy] explode all trees

#2 (abstinen*OR dependen* or addict* or withdraw* or misus*OR abus*):ti,ab,kw (Word variations have been searched)

#3 #1 or #2

#4 amphetamine or amfetamine or methamphetamine or mdma or ecstasy or dextroamphetamine or stimulant or stimulants:ti,ab,kw (Word variations have been searched)

#5 #3 and #4

#6 MeSH descriptor: [Cognitive Therapy] explode all trees

#7 CBT:ab,ti

#8 (cogniti* near/3 (behavio* or therap*)):ab,ti

#9 (cogniti* near/3 (technique* or restructur* or challeng*)):ab,ti

#10 #6 OR #7 OR #8 OR #9

#11 #5 AND #10

**3 MEDLINE search strategy**

**MEDLINE (via PubMed)**

**2 July 2018 (287 hits)**

1. Substance-Related Disorders [MeSH]
2. (abstinen*[tiab] OR dependen*[tiab] OR addict*[tiab] OR withdraw*[tiab] OR misus*[tiab] OR use*[tiab] OR abus*[tiab])
3. #1 OR #2
4. Amphetamines[MeSH]
5. (amphetamine[tiab] OR amfetamine[tiab] OR methamphetamine[tiab] OR MDMA[tiab] OR ecstasy[tiab] OR dextroamphetamine[tiab] OR stimulant[tiab] OR stimulants[tiab])
6. #4 OR #5
7. cognitive therapy[MeSH]
8. CBT[tiab]
9. (cogniti*[tiab] AND (behavio*[tiab] OR therap*[tiab]))
10. (cogniti*[tiab] AND (technique* [tiab] OR restructur*[tiab] OR challeng*[tiab]))
11. #7 OR #8 OR #9 OR #10
12. randomized controlled trial [pt]
13. controlled clinical trial [pt]
14. placebo [tiab]
15. drug therapy [sh]
16. randomly [tiab]
17. trial [tiab]
18. groups [tiab]
19. #12 OR #12 OR #13 OR #14 OR #15 OR #16 OR #17 OR #18
20. animals [mh] NOT humans [mh]
21. #19 NOT #20
22. #3 AND #6 AND #11 AND #21

**4 Embase search strategy**

**2 July 2018 (276 hits)**

#1 drug dependence'/exp OR 'drug abuse'/exp OR 'substance abuse'/exp

#2 abstinen*:ab,ti OR dependen*:ab,ti OR addict*:ab,ti OR withdraw*:ab,ti OR misus*:ab,ti OR abus*:ab,ti

#3 #1 OR #2

#4 'amphetamine derivative'/exp

#5 amphetamine:ab,ti OR amfetamine:ab,ti OR methamphetamine:ab,ti OR mdma:ab,ti OR ecstasy:ab,ti OR dextroamphetamine:ab,ti ORstimulant:ab,ti OR stimulants:ab,ti

#6 #4 OR #5

#7 #3 AND #6

#8 cognitive therapy'/exp

#9 cbt:ab,ti

#10 (cogniti* NEAR/3 (behavio* OR therap*)):ab,ti

#11 (cogniti* NEAR/3 (technique* OR restructur* OR challeng*)):ab,ti

#12 #8 OR #9 OR #10 OR #11

#13 #7 AND #12

#14 crossover procedure'/exp OR 'double blind procedure'/exp OR 'single blind procedure'/exp OR 'controlled clinical trial'/exp OR'clinical trial'/exp OR 'randomized controlled trial'/exp OR placebo:ab,ti OR 'double blind':ab,ti OR 'single blind':ab,ti OR assign*:ab,ti OR allocat*:ab,ti OR volunteer*:ab,ti OR random*:ab,ti OR factorial*:ab,ti OR crossover:ab,ti OR (cross:ab,ti AND over:ab,ti)

#15 #13 AND #14

**5 Web of Science search strategy**

**WOS (via THOMSON REUTERS)**

**2 July 2018 (74 hits)**

#1 TS= clinical trial* OR TS=research design OR TS=comparative stud* OR TS=evaluation stud* OR TS=controlled trial* OR TS=follow-up stud* OR TS=prospective stud* OR TS=random* OR TS=placebo* OR TS=(single blind*) OR TS=(double blind*)

#2 TS=((amphetamine OR amfetamine OR methamphetamine OR mdma OR ecstasy OR dextroamphetamine OR stimulant OR stimulants) NEAR/6 (abstinen OR dependen* OR addict* OR withdraw* OR misus* OR abus*))

#3 TI=CBT

#4 TS=(cogniti* NEAR/3 (behavio* OR therap*))

#5 TS=(cogniti* NEAR/3 (technique* OR restructur* OR challeng*))

#6 #3 OR #4 OR #5

#7 #1 OR #2 OR #6

**6 PsycINFO search strategy**

**2 July 2018 (129 hits)**

1. Clinical Trials.sh.
2. Placebo.sh.
3. placebo$.ti,ab.
4. randomly.ab.
5. randomi#ed.ti,ab.
6. trial.ti,ab.
7. ((singl$ or doubl$ or trebl$ or tripl$) adj3 (blind$ or mask$ or dummy)).mp.
8. (control$ adj3 (trial$ or study or studies or group$)).ti,ab.
9. factorial$.ti,ab.
10. allocat$.ti,ab.
11. assign$.ti,ab.
12. volunteer$.ti,ab.
13. (crossover$ or cross over$).ti,ab.
14. (quasi adj (experimental or random$)).mp.
15. 1 or 2 or 3 or 4 or 5 or 6 or 7 or 8 or 9 or 10 or 11 or 12 or 13 or 14
16. exp cognitive therapy/
17. CBT.ti,ab.
18. (cogniti* adj3 (behavio* or therap*)).mp.
19. (cogniti* adj3 (technique* or restructur* or challeng*)).mp.
20. 16 or 17 or 18 or 19
21. (amphetamine or amfetamine or methamphetamine or mdma or ecstasy or dextroamphetamine or stimulant or stimulants).mp.
22. exp drug dependency/ or exp drug addiction/
23. (addict* or abus* or abstain* or abstinen* or dependen* or disorder* or misuse*).mp.
24. 22 or 23
25. 21 and 24
26. 15 and 20 and 25

**7 Assessment of risk of bias in included studies**

**1. Sequence generation (checking for possible selection bias)**

For each included trial, we described the method used to generate the allocation sequence in sufficient detail to allow an assessment of whether it should produce comparable groups. We assessed the method as:

- low risk of bias (any truly random process, e.g. random number table; computer random number generator);
- high risk of bias (any non-random process, e.g. odd or even date of birth; hospital or clinic record number) or
- unclear risk of bias (insufficient information to permit judgement).

**2. Allocation concealment (checking for possible selection bias)**

For each included trial, we described the method used to conceal the allocation sequence and determine whether intervention allocation could have been foreseen in advance of, or during, recruitment, or changed after assignment. We assessed the methods as:

- low risk of bias (e.g. telephone or central randomisation; consecutively numbered sealed opaque envelopes);
- high risk of bias (e.g. open random allocation; unsealed or non-opaque envelopes, alternation; date of birth) or
- unclear risk of bias (insufficient information to permit judgement).

**3.1. Blinding of participants and personnel (checking for possible performance bias)**

Blinding of personnel delivering the intervention and participants is not feasible. It is not desirable to blind participants to the knowledge of which intervention they are receiving. Knowledge that you are participating in a cognitive-behavioural programme is part of the intervention (this knowledge is often categorised along with other non-specific intervention factors). For this reason, we did not assess the risk of bias of this item.

**3.2. Blinding of outcome assessment (checking for possible detection bias)**

For each included trial, we described the methods used, if any, to blind outcome assessors from the knowledge of which intervention a participant received. We assessed blinding separately for different outcomes or classes of outcomes. Outcomes were grouped into subjective (drug use measured by urine analysis, dropout rate, death) and objective (self-reported drug use, psychological outcomes).

We assessed methods used to blind outcome assessment as:

Objective outcomes:

- low risk of bias: no blinding of outcome assessment, but the review authors judged that the outcome measurement was unlikely to be influenced by lack of blinding; blinding of outcome assessment ensured and unlikely that the blinding could have been broken;
- high risk of bias: no blinding of outcome assessment and the outcome measurement was likely to be influenced by lack of blinding; blinding of outcome assessment, but likely that the blinding could have been broken and the outcome measurement was likely to be influenced by lack of blinding or
- unclear risk of bias: insufficient information to permit judgement.

Subjective outcomes:

- low risk of bias: blinding of outcome assessment ensured, and unlikely that the blinding could have been broken;
- high risk of bias: no blinding of outcome assessment, and the outcome measurement was likely to be influenced by lack of blinding; blinding of outcome assessment, but likely that the blinding could have been broken, and the outcome measurement was likely to be influenced by lack of blinding or
- unclear risk of bias: insufficient information to permit judgement.

**4. Incomplete outcome data (checking for possible attrition bias through withdrawals, dropouts, protocol deviations)**

For each included trial and for each outcome or class of outcomes, we described the completeness of data including attrition and exclusions from the analysis. We stated whether attrition and exclusions were reported, the numbers included in the analysis at each stage (compared with the total number of randomised participants), reasons for attrition or exclusion where reported, and whether missing data were balanced across groups or were related to outcomes. Where sufficient information was reported, or could be supplied by the trial authors, we reincluded missing data in the analyses we undertook. We assessed methods as:

- low risk of bias: e.g. less than 20% missing outcome data; missing outcome data balanced across groups;
- high risk of bias: e.g. numbers or reasons for missing data imbalanced across groups; 'as treated' analysis done with substantial departure of intervention received from that assigned at randomisation or
- unclear risk of bias: insufficient information to permit judgement.

**5. Selective reporting bias**

For each included trial, we described how we investigated the possibility of selective outcome reporting bias and our findings. We assessed the methods as:

- low risk of bias: where it was clear that all of the trial's prespecified outcomes and all expected outcomes of interest to the review were reported;
- high risk of bias: where not all the trial's prespecified outcomes were reported; one or more reported primary outcomes were not prespecified; outcomes of interest were reported incompletely and so could not be used; trial failed to include results of a key outcome that would have been expected to have been reported) or
- unclear risk of bias: insufficient information to permit judgement.
